# Association Between Proton Pump Inhibitors and Asthma: A Population-Based Cohort Study

**DOI:** 10.3389/fphar.2020.00607

**Published:** 2020-05-08

**Authors:** Yao-Tung Wang, Ming-Chang Tsai, Yu-Hsun Wang, James Cheng-Chung Wei

**Affiliations:** ^1^Institute of Medicine, Chung Shan Medical University, Taichung, Taiwan; ^2^School of Medicine, Chung Shan Medical University, Taichung, Taiwan; ^3^Division of Pulmonary Medicine, Department of Internal Medicine, Chung Shan Medical University Hospital, Taichung, Taiwan; ^4^Division of Gastroenterology and Hepatology, Department of Internal Medicine, Chung Shan Medical University Hospital, Taichung, Taiwan; ^5^Department of Medical Research, Chung Shan Medical University Hospital, Taichung, Taiwan; ^6^Beijing Tsinghua Changgung Hospital, School of Clinical Medicine, Tsinghua University, Beijing, China; ^7^Division of Allergy, Immunology and Rheumatology, Department of Internal Medicine, Chung Shan Medical University Hospital, Taichung, Taiwan; ^8^Graduate Institute of Integrated Medicine, China Medical University, Taichung, Taiwan

**Keywords:** proton pump inhibitors, asthma, National Health Insurance Research Database, population base study, histamine 2 receptor antagonists

## Abstract

**Objective:**

The relationship between proton pump inhibitors (PPIs) and asthma is controversial. The goal of this study was to determine the association between PPI use in non-asthma subjects and their subsequent asthma prevalence.

**Design:**

Nationwide, population-based cohort study.

**Methods:**

We conducted a nationwide, population-based retrospective cohort study using data from the National Health Insurance Research Database (NHIRD) of Taiwan from 1999 to 2013. We identified 24,077 adult patients with PPI use for more than 3 months and 24,077 controls matched by propensity score on a one-to-one ratio for age, gender, comorbidities (hypertension, hyperlipidemia, gastroesophageal reflux disease, allergic rhinitis, atopic dermatitis, peptic ulcer disease, diabetes, and sleep apnea syndrome), and medications (histamine 2 receptor antagonists [H2RA], nonsteroidal anti-inflammatory drugs [NSAIDs], and acetaminophen). The cumulative asthma incidence for the two cohorts in the follow-up period was estimated with the Kaplan–Meier method, and the difference was examined using the log-rank test. Multivariate Cox regression models were used to calculate the adjusted hazard ratios (HR).

**Results:**

The overall incidence of asthma was 1.58-fold greater in the PPI cohort than in the non-PPI cohort (13.3 *versus* 8.4 per 1,000 person-years), with an adjusted HR of 1.76 (95% confidence interval [CI], 1.64–1.88). In patients without previous peptic ulcer disease, the adjusted HR of asthma associated with PPIs was higher than in the non-PPI group (1.95; 95% CI, 1.80–2.11). The risk of asthma due to PPI use was also more significant in patients not receiving H2RA (1.81; 95% CI, 1.66–1.96), NSAIDs (1.93; 95% CI, 1.73–2.15), and acetaminophen (1.88; 95% CI, 1.70–2.08).

**Conclusions:**

This population base study demonstrated that patients with long-duration of PPI use are at a higher risk of developing asthma, regardless of age, gender, comorbidities, and medications.

## Highlights

1. What is already known about this topic?
  The relationship between proton pump inhibitors (PPIs) and asthma is controversial. Proton pump inhibitors are the most common acid-suppressive medications of GERD which can contribute to asthma symptoms or has been identified as a potential trigger for asthma. Increasing numbers of studies have suggested PPIs are associated with significant rise of adverse health outcomes.
2. What does this article add to our knowledge?
  Our data suggest that patients with long-time PPI use have a significantly higher risk of developing asthma when compared with the general population, regardless of gender and age.
3. How does this study impact current management guidelines?
  The higher risk of developing asthma of patients with long-duration PPI use is identified based on the general population. We recommended H2RA use prior to PPIs in patients who have no underlying peptic ulcers.

## Introduction

Clinically, gastroesophageal reflux disease (GERD) is often reported to be a common cause of chronic cough (Kastelik et al., 2005; Irwin et al., 2006). GERD can also contribute to asthma symptoms or has been identified as a potential trigger for asthma (Depla et al., 1988; Pope, 1994; Harding, 2003; Chipps et al., 2018). The cause may be an aspiration of acid or gastric contents into airways and alveoli, which then leads to stimulation of receptors in the lower respiratory tract or chronic inﬂammation and damage to the alveolar-capillary membrane (Ravelli et al., 2006). GERD and asthma often coexist: GERD has a prevalence ranging from 30 to 90% in persons with asthma compared to an average of 24% in non-asthmatic controls (Bor et al., 2010; Broers et al., 2018).

GERD treatments include the use of proton pump inhibitors (PPIs), which are the most common acid-suppressive medications (Forgacs and Loganayagam, 2008) and the first-line antisecretory therapy (Wolfe and Sachs, 2000). PPIs are often used for empiric management of chronic cough with a suspected relationship to GERD (Kahrilas et al., 2016); indeed, two placebo-controlled trials have suggested that PPI treatment relieves GERD-related chronic cough (Harding, 2003). Many studies have been conducted to evaluate and confirm the efficacy of PPI therapy on asthma outcome in patients with asthma with or without GERD. For example, aggressive acid suppressive therapy with omeprazole for 3 months improved asthma symptoms and pulmonary function in 73% of asthmatics with GERD (Meier et al., 1994).

PPI is a key component in the treatment of chronic cough due to GERD and an optimum regimen to improve asthma symptoms and pulmonary function in select patients. The aim of this study was to identify the association between PPI use in non-asthma subjects and their subsequent asthma prevalence.

## Methods

### Data Resources

This study is a nationwide, population-based retrospective cohort study based on the Taiwan National Health Insurance Research Database (NHIRD) of one million patients between January1, 1999 and December 31, 2013. The NHIRD, established by the Bureau of National Health Insurance (BNHI) and the National Health Research Institutes (NHRI), included health care data that covered more than 99% of the Taiwanese population (approximately 23 million people) (https://www.nhi.gov.tw/english/Default.aspx). For patient privacy, the patient identity numbers are encrypted by the NHIRD. The database is a record of all the insurance information, such as medical records, and includes disease diagnosis, drug prescriptions (order date and duration, drug name, dosage, frequency, and administration route), medical procedures, and date of clinic visits and hospitalizations (Lee et al., 2010). The diseases in the NHIRD were defined according to the International Statistical Classification of Diseases and Related Health Problems-9th Edition, clinical modification (ICD-9-CM) codes.

This study was approved by the Institutional Review Board (IRB) of Chung Shan Medical University Hospital, Taiwan (CS15134). The IRB waived the need for informed consent for this retrospective study, based on the NHIRD. All protocols were performed in accordance with the relevant guidelines and regulations and were under the surveillance of the IRB of Chung Shan Medical University Hospital.

### Study Population

The new PPI users were selected from one million random samples from the NHIRD between 2000 and 2012 by searching for use of the PPI drugs omeprazole, esomeprazole, lansoprazole, pantoprazole, and rabeprazole for more than 90 days and patient age more than twenty years. The index date was operationalized as the first date of PPI use. The outcome of the analysis was a diagnosis of asthma. The asthma cases were included from the same random samples using the codes ICD-9-CM 493.XX and included admitted inpatients or outpatients with at least two visits to the outpatient department clinics. The asthma-related comorbidities of hypertension patients were identified using the ICD-9-CM codes 401-405, and hyperlipidemia was identified with the ICD-9-CM codes 272.0-272.4. Other selected conditions included GERD (ICD-9-CM codes 530.11 and 530.81), allergic rhinitis (ICD-9-CM codes 472, 473 and 477), atopic dermatitis (ICD-9-CM codes 691), peptic ulcer disease (ICD-9-CM codes 531-534.9), diabetes (ICD-9-CM codes 250), and sleep apnea syndrome (ICD-9-CM codes 327.23, 780.51, 780.52, 780.53, and780.57).

**Figure 1:**
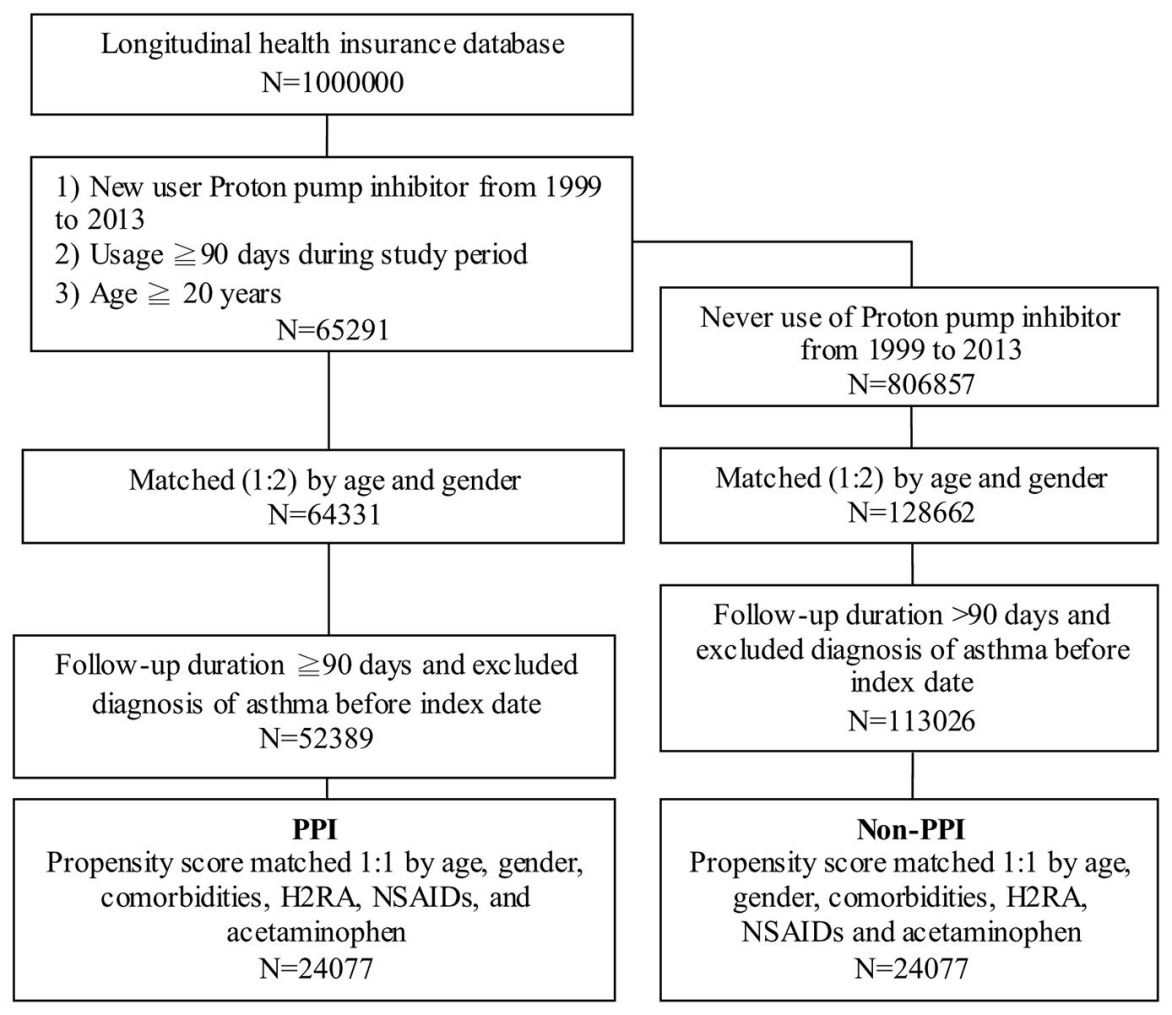
Case selection flow chart of patient selection from one million nationally representative patients in the Taiwan National Health Insurance Research Database PPI, proton pump inhibitor; H2RA, histamine 2 receptor antagonists; NSAIDs, nonsteroidal anti-inflammatory drugs.

In the treatment cohort, 65,291 new PPI users were enrolled, and 64,331 patient samples were obtained after matching to 128,662 non-PPI users in the control group by age and gender in 1:2 ratios (**Figure 1**). After a follow-up duration of more than ninety days and excluding the asthma cases diagnosed before the index date, 52,389 PPI users in the case group and 113,026 non-PPI patients in the control group were enrolled. Patients who had been diagnosed with asthma prior to the use of PPIs were excluded to ensure that a causal relationship could be analyzed in this study.

The PPI case group and the non-PPI control group also were matched by propensity score on a one-to-one ratio for age, gender, comorbidities (*e.g.*, hypertension, hyperlipidemia, GERD, allergic rhinitis, atopic dermatitis, peptic ulcer disease, diabetes, and sleep apnea syndrome), and medications (*e.g.*, histamine 2 receptor antagonists [H2RA], nonsteroidal anti-inflammatory drugs [NSAIDs], and acetaminophen). Ultimately, 24,077 subjects were included in the PPI group and 24,077 in the non-PPI group.

### Data Processing and Statistical Analysis

We used the Chi-square test for categorical variables and t-tests for continuous variables to analyze the differences in the demographic data between the PPI and non-PPI groups. The Kaplan–Meier method was used to estimate the cumulative asthma incidence for the two cohorts in the follow-up period and the log-rank test was used to examine the difference. Multivariate Cox regression models were used to calculate the adjusted hazard ratios (HRs, including the 95% confidence intervals [CIs]) of the PPI group for non-PPI group after adjusting for age, gender, hypertension, hyperlipidemia, GERD, allergic rhinitis, peptic ulcer disease, diabetes, sleep apnea syndrome, H2RA, NSAIDs, and acetaminophen. All data analyses were performed using SPSS 18.0 (SPSS Inc., Chicago, IL, USA). A two-tailed P-value of 0.05 was considered statistically significant in this study.

## Results

The study cohorts consisted of 24,077 matched PPI patients and 24,077 matched non-PPI control subjects. The age and gender distributions were similar in both cohorts (**Table 1**). Most subjects were 40–65 years of age (53.8% of PPI and 53.3% of non-PPI). The mean ages of the PPI and non-PPI cohorts were 55.8 ± 15.5 and 56.7 ± 15.1 years, respectively.

**Table 1:** Demographic characteristics, comorbidity, and medication in patients with asthma using or not using proton pump inhibitors (PPIs).

	Unmatched					Matched				
	PPI (N = 52,389)		Non-PPI (N = 113,026)			PPI (N = 24,077)		Non-PPI (N = 24,077)		
	N	%	n	%	p-value	n	%	n	%	p-value
Age					0.006*					0.168
20–40	9,390	17.9	19,774	17.5		3,818	15.9	3,745	15.6	
40–65	28,632	54.7	61,485	54.4		12,959	53.8	12,845	53.3	
>=65	14,367	27.4	31,767	28.1		7,300	30.3	7,487	31.1	
Mean ± SD	54.8 ± 15.4		55.1 ± 15.4		0.001*	55.8 ± 15.5		56.7 ± 15.1		<0.001*
Gender					0.076					0.784
Male	23,705	45.2	51,670	45.7		13,135	54.6	13,105	54.4	
Female	28,684	54.8	61,356	54.3		10,942	45.4	10,972	45.6	
Hypertension	15,915	30.4	22,644	20.0	<0.001*	7,429	30.9	7,533	31.3	0.306
Hyperlipidemia	7,270	13.9	8,618	7.6	<0.001*	3,171	13.2	3,247	13.5	0.308
GERD	5,418	10.3	344	0.3	<0.001*	341	1.4	344	1.4	0.908
Allergic rhinitis	4,439	8.5	4,764	4.2	<0.001*	1,740	7.2	1,802	7.5	0.279
Atopic dermatitis	359	0.7	555	0.5	<0.001*	157	0.7	159	0.7	0.910
Peptic ulcer disease	26,648	50.9	3,333	2.9	<0.001*	3,336	13.9	3,333	13.8	0.968
Diabetes	8,549	16.3	9,774	8.6	<0.001*	3,748	15.6	3,798	15.8	0.531
Sleep apnea syndrome	3,209	6.1	2,983	2.6	<0.001*	1,274	5.3	1,289	5.4	0.761
H2RA	19,240	36.7	6,228	5.5	<0.001*	5,786	24.0	5,758	23.9	0.765
NSAIDs	25,288	48.3	33,473	29.6	<0.001*	10,818	44.9	11,003	45.7	0.090
Acetaminophen	23,402	44.7	23,927	21.2	<0.001*	9,476	39.4	9,625	40.0	0.165

*p < 0.01.

PPI, proton pump inhibitor; GERD, gastroesophageal reflux disease; H2RA, histamine 2 receptor antagonists; NSAIDs, nonsteroidal anti-inflammatory drugs.

**Figure 2:**
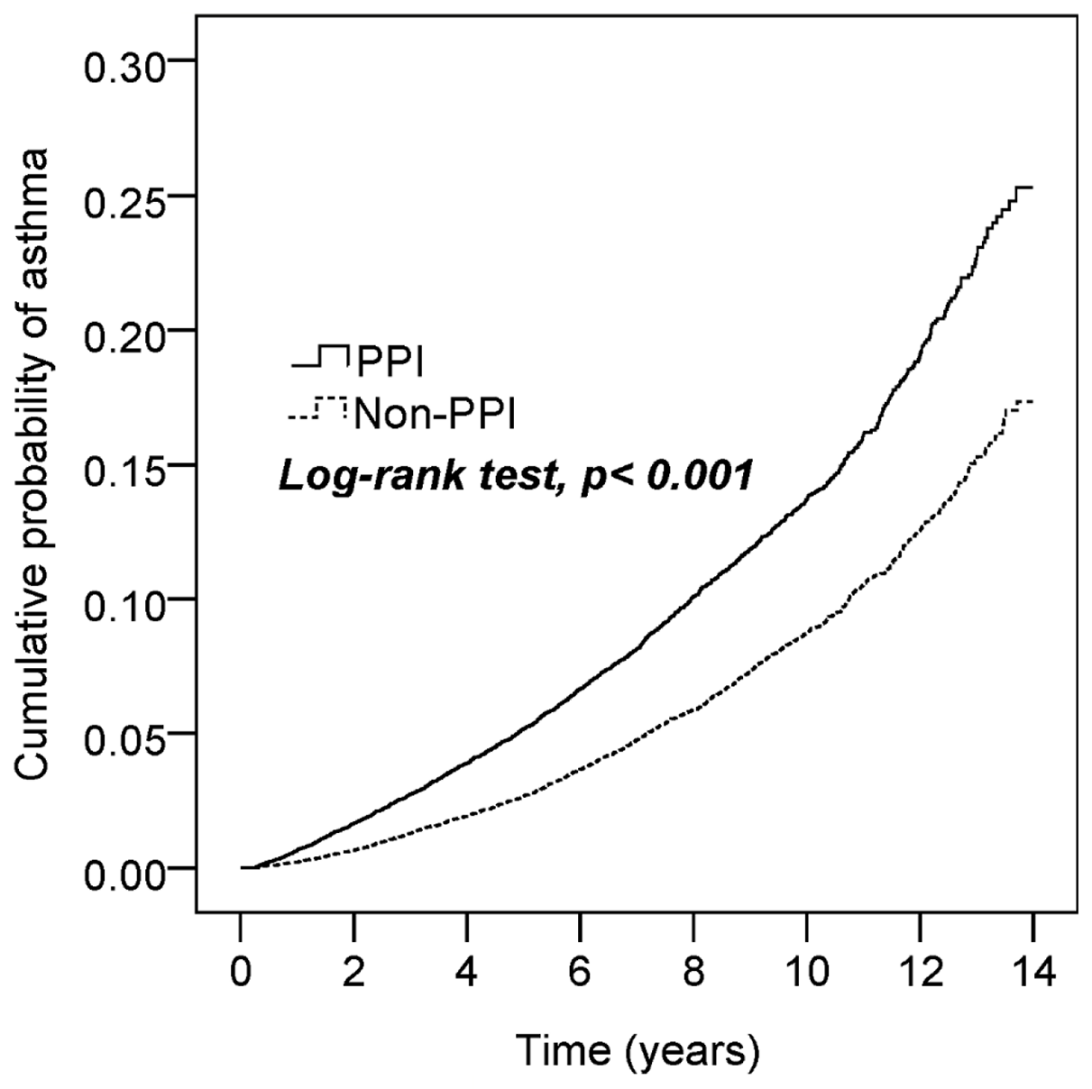
Cumulative incidence of asthma in patients with and without proton pump inhibition (PPI) use.

The comorbidities, including allergic rhinitis, atopic dermatitis, GERD, peptic ulcer disease, hypertension, hyperlipidemia, diabetes, sleep apnea syndrome, and use of H2RA, NSAIDs, and acetaminophen, were significantly different between the unmatched PPI patients and the non-PPI controls (*p-values <0.001*), but no difference was observed between the matched PPI and non-PPI groups. The average follow-up period was 5.7 ± 3.4 years (137,582 person-years) for the PPI cohort and 6.9 ± 3.5 years (165,323 person-years) for the non-PPI cohort. Kaplan–Meier analysis showed that the cumulative probability of developing asthma by the end of the 14-year follow-up period was higher for the PPI cohort than for the non-PPI cohort (log-rank test, *p < 0.001*; **Figure 2**).

**Table 2** shows a total of 3,216 asthma events (1,823 for the PPI cohort and 1,393 for the non-PPI cohort). The incidence was 1.70-fold higher (95% CI, 1.59–1.83) in the PPI cohort than in the non-PPI cohort (13.3 *vs.* 8.4 per 1,000 person-years), with an adjusted HR of 1.76 (95% CI, 1.64–1.88) after controlling for age, gender, comorbidities, and medications. The incidence of asthma decreased with age in both cohorts (*p =0.019*), but the age-specific adjusted HRs for the PPI cohort, when compared with the non-PPI cohort, were statistically significant for all age groups. Among the analysis of the listed comorbidities, we found that the asthma prevalence was higher for patients without peptic ulcer disease in the PPI cohort (adjusted HR of 1.95; 95% CI, 1.80–2.11) but did not differ in patients with peptic ulcer disease (adjusted HR of 1.14; 95% CI, 0.97–1.34). Patients not using H2RA before PPIs had a higher incidence of asthma (adjusted HR of 1.81; 95% CI, 1.66–1.96) than in those cases who had used H2RA (adjusted HR of 1.58; 95% CI, 1.38–1.79). If the patients were not exposed to asthma-related medications (NSAIDs and acetaminophen), the asthma risks due to PPI use were also higher (adjusted HR of 1.93; 95% CI, 1.73–2.15 in the NSAIDs cohort and 1.88; 95% CI, 1.70–2.08 in the acetaminophen cohort).

**Table 2:** Incidence of asthma and the Cox proportional hazard model estimated proton pump inhibitor (PPI) cohort to non-PPI cohort hazard ratio.

	PPI			Non-PPI				
	Asthma event	PY	rate	Asthma event	PY	rate	Crude HR (95% C.I.)	Adjusted HR (95% C.I.)
Total	1,823	137,582	13.3	1,393	165,323	8.4	1.70 (1.59–1.83)	1.76 (1.64–1.88)
Age								
20–40	196	23,859	8.2	113	27,634	4.1	2.21 (1.75–2.79)	2.22 (1.76–2.80)
40–65	891	77,631	11.5	643	91,818	7.0	1.81 (1.63–2.00)	1.83 (1.66–2.03)
>=65	736	36,091	20.4	637	45,870	13.9	1.56 (1.41–1.74)	1.60 (1.44–1.78)
								p for interaction = 0.019
Gender								
Male	894	75,156	11.9	721	90,282	8.0	1.61 (1.46–1.78)	1.65 (1.50–1.83)
Female	929	62,426	14.9	672	75,041	9.0	1.80 (1.63–1.99)	1.86 (1.69–2.06)
								p for interaction= 0.127
Hypertension								
No	1,187	100,312	11.8	839	119,430	7.0	1.86 (1.70–2.03)	1.90 (1.74–2.08)
Yes	636	37,270	17.1	554	45,893	12.1	1.50 (1.34–1.68)	1.55 (1.38–1.74)
								p for interaction = 0.009
Hyperlipidemia								
No	1,590	121,675	13.1	1,206	146,560	8.2	1.73 (1.60–1.86)	1.78 (1.65–1.92)
Yes	233	15,908	14.6	187	18,763	10.0	1.54 (1.27–1.87)	1.60 (1.32–1.94)
								p for interaction = 0.354
GERD								
No	1,803	136,289	13.2	1,380	163,997	8.4	1.70 (1.59–1.83)	1.76 (1.64–1.89)
Yes	20	1,293	15.5	13	1,326	9.8	1.57 (0.78–3.16)	1.57 (0.77–3.20)
								p for interaction = 0.786
Allergic rhinitis								
No	1,652	128,132	12.9	1,236	153,854	8.0	1.74 (1.62–1.87)	1.80 (1.67–1.94)
Yes	171	9,450	18.1	157	11,469	13.7	1.41 (1.14–1.75)	1.44 (1.15–1.79)
								p for interaction = 0.065
Atopic dermatitis								
No	1,806	136,762	13.2	1,380	164,317	8.4	1.70 (1.59–1.83)	1.80 (1.68–1.93)
Yes	17	820	20.7	13	1,006	12.9	1.70 (0.82–3.50)	1.76 (0.84–3.69)
								p for interaction = 0.908
Peptic ulcer disease								
No	1,521	117,586	12.9	1,089	143,531	7.6	1.87 (1.73–2.02)	1.95 (1.80–2.11)
Yes	302	19,996	15.1	304	21,792	13.9	1.11 (0.95–1.30)	1.14 (0.97–1.34)
								p for interaction < 0.001
Diabetes								
No	1,547	120,310	12.9	1,163	142,852	8.1	1.72 (1.59–1.85)	1.76 (1.63–1.90)
Yes	276	17,272	16.0	230	22,471	10.2	1.66 (1.39–1.97)	1.72 (1.44–2.05)
								p for interaction = 0.943
Sleep apnea syndrome								
No	1,722	131,137	13.1	1,298	157,665	8.2	1.73 (1.61–1.86)	1.78 (1.66–1.92)
Yes	101	6,445	15.7	95	7,658	12.4	1.32 (0.997–1.75)	1.33 (1.002–1.76)
								p for interaction = 0.074
H2RA								
No	1,292	97,235	13.3	983	119,937	8.2	1.75 (1.61–1.90)	1.81 (1.66–1.96)
Yes	531	40,347	13.2	410	45,386	9.0	1.56 (1.37–1.77)	1.58 (1.38–1.79)
								p for interaction = 0.029
NSAIDs								
No	803	61,456	13.1	560	75,846	7.4	1.84 (1.65–2.05)	1.93 (1.73–2.15)
Yes	1,020	76,126	13.4	833	89,477	9.3	1.58 (1.44–1.73)	1.61 (1.47–1.76)
								p for interaction < 0.001
Acetaminophen								
No	931	72,357	12.9	626	85,788	7.3	1.82 (1.65–2.02)	1.88 (1.70–2.08)
Yes	892	65,225	13.7	767	79,535	9.6	1.58 (1.44–1.74)	1.64 (1.49–1.81)
								p for interaction = 0.001

PY, Person-years; Rate, 1/1,000 Person-Years; PPI, proton pump inhibitor; GERD, gastroesophageal reflux disease; H2RA, histamine 2 receptor antagonists; NSAIDs, nonsteroidal anti-inflammatory drugs.

**Table 3** shows the results of the Cox proportional hazard model estimated PPI hazard ratio among the different PPI types. Esomeprazole, lansoprazole, omeprazole, and pantoprazole were significantly related to asthma risk, but rabeprazole was not (adjusted HR of 1.04; 95% CI, 0.94–1.15).

**Table 3:** Cox proportional hazard model estimated proton pump inhibitor (PPI) hazard ratio among different PPI types.

	N	No. of asthma event	Crude HR	95% C.I.	Adjusted HR^†^	95% C.I.
Esomeprazole	12,807	1,071	1.27*	1.17–1.38	1.26*	1.15–1.37
Lansoprazole	13,842	1,187	1.18*	1.08–1.28	1.23*	1.13–1.34
Omeprazole	10,530	1,054	1.36*	1.25–1.49	1.38*	1.26–1.50
Pantoprazole	10,131	841	1.15*	1.05–1.26	1.13*	1.04–1.24
Rabeprazole	5,705	448	1.01	0.91–1.12	1.04	0.94–1.15

^†^Adjusted for age, gender, hypertension, hyperlipidemia, GERD, allergic rhinitis, atopic dermatitis, peptic ulcer disease, diabetes, sleep apnea syndrome, H2RA, NSAIDs, and acetaminophen.; *p < 0.01.

## Discussion

To our knowledge, this is the first study to evaluate the relationship between PPI use and the subsequent risk of asthma. We identified a significant risk of asthma among patients using PPIs when compared to the non-PPI population after matching by propensity score and adjusting for age, gender, comorbidities, and asthma medications. The incidence of asthma was higher in patients not using H2RA before PPIs than in those cases who had used H2RA. Patients not exposed to asthma-related medications (NSAIDs and acetaminophen) also had a higher asthma risk due to PPIs (adjusted HR of 1.93 in the NSAIDs cohort and 1.88 in the acetaminophen cohort).

Many studies have proved that some medications, like aspirin, NSAIDs, and acetaminophen, may be related to the risk of asthma. The use of acetaminophen may represent an important risk factor for the development and/or maintenance of asthma in adolescent children (Beasley et al., 2011). For example, paracetamol exposure in pregnancy and early infancy has been considered to have an association with the subsequent development of childhood asthma (age ≥ 5 years) (Cheelo et al., 2015). A previous retrospective cohort study of the Taiwan NHIRD indicated that short-term aspirin, ibuprofen, and diclofenac use was probably correlated with asthma exacerbation in asthmatic children (Lo et al., 2016). However, none of these studies mentioned any possible association of acid-suppressive therapy and asthma risk. The fully adjusted Cox regression model presented in our study for competing risk analysis (*i.e.*, age, gender, allergic rhinitis, atopic dermatitis, GERD, peptic ulcer disease, hypertension, hyperlipidemia, diabetes, sleep apnea syndrome, and use of H2RA, NSAIDs, and acetaminophen) suggests that PPIs may play an independent role in the development of asthma.

PPIs inhibit the H^+^-K^+^-ATPase, which is the final step of gastric acid secretion by parietal cells. Since the 1980s, when the first generation of PPIs was made available, PPIs have become the most commonly used medications for acid suppression (Forgacs and Loganayagam, 2008). PPIs are the first-line anti-secretory therapy in the treatment of peptic ulcer disease and are indicated as a treatment for GERD, including erosive esophagitis, and as maintenance therapy in patients with severe erosive esophagitis or Barrett’s esophagus (Wolfe and Sachs, 2000). PPIs are also effective in preventing NSAID-induced gastroduodenal toxicity and in healing gastroduodenal ulcers associated with NSAIDs when NSAIDS cannot be discontinued (Agrawal et al., 2000). In the COGENT trial, prophylactic use of a PPI reduced the rate of upper gastrointestinal bleeding among patients receiving aspirin and clopidogrel (Bhatt et al., 2010).

In recent years, increasing numbers of studies have suggested an association between PPIs and an increased risk of adverse health outcomes. Some studies have shown that PPIs are associated with a significant rise in acute interstitial nephritis (Blank et al., 2014), chronic kidney disease (CKD) (Lazarus et al., 2016), kidney disease progression, and end-stage renal disease (Xie et al., 2017). A large prospective observational German cohort study showed that patients receiving PPIs had a higher risk of incident dementia (Gomm et al., 2016). PPI use has been associated with an increased risk of incident and recurrent *Clostridium difficile* infections (Kwok et al., 2012), community-acquired pneumonia (Filion et al., 2014), and hospital-associated pneumonia (Herzig et al., 2009). A relationship between cardiovascular events and PPI use has also been mentioned (Melloni et al., 2015). A longitudinal observational cohort study of United States veterans also found an increased risk of death among users of PPIs (Xie et al., 2017). Our study found that PPI use is an independent risk for asthma.

Aggressive acid suppressive therapy with PPIs has been recommended to improve asthma outcomes in asthmatics with GERD. A study of 30 nonsmoking adult asthmatics with GERD found that a 3-month regimen of acid suppressive therapy with omeprazole improved asthma symptoms and pulmonary function in 73% of the subjects (Harding et al., 1996). Two placebo-controlled trials that investigated the efficacy of PPI on GERD-related chronic cough indicated that PPI treatment relieves GERD-related chronic cough but recommended using a double-standard dose of the PPI for a minimum of 2 to 3 months (Harding, 2003). However, a parallel-group, double-blind trial of 412 participants with asthma inadequately controlled by inhaled corticosteroids and with minimal or no symptoms of GERD showed no improvement in asthma outcomes by a treatment with 40 mg esomeprazole twice a day; these findings indicated that asymptomatic GERD might not be a possible cause of poorly controlled asthma (Mastronarde et al., 2009).

A review study identified 13 publications from 1989 to 2012 related to treatment of asymptomatic GERD in school-age children with asthma poorly controlled by inhaled corticosteroids. The FDA-approved doses of PPIs did not improve their asthma outcomes (Blake and Teague, 2013); consequently, the authors commented that GERD and asthma may be associated by chance alone, because GERD is highly prevalent in the general population (Dent et al., 2005).

PPIs provide a stronger acid suppression when compared to H2RA, so they result in a faster control of peptic ulcer disease symptoms and higher ulcer healing rates (Walan et al., 1989). A meta-analysis that enrolled fourteen trials and a total of 1,720 patients concluded that PPIs were more effective than H2RA at reducing clinically important and overt upper gastrointestinal bleeding. No differences were noted between PPIs and H2RA in the risk of nosocomial pneumonia, ICU mortality, or ICU length of stay (Alhazzani et al., 2013). However, a decision-analytic model aimed at determining the cost effectiveness of stress ulcer prophylaxis with H2RA versus PPIs in critically ill and mechanically ventilated adults from a health care institutional perspective concluded that providing stress ulcer prophylaxis with H2RA therapy may reduce costs, increase survival, and avoid complications when compared with PPI therapy (Hammond et al., 2017).

A recent multicenter retrospective study examined the effect of preventing clinically important GI bleeding (CIGIB) with prophylactic PPIs or H2RA among critically ill adults with at least one stress ulcer risk factor in ICU care in US nonfederal hospitals. They found the hazard ratio for CIGIB was two times greater for PPIs patients than for H2RA patients (adjusted HR 1.82 [95% CI, 1.19–2.78]) (Lilly et al., 2018). H2RA administration has been associated with many rare side effects (cardiac arrhythmia; cardiac arrest occurring with rapid infusion (Hinrichsen et al., 1995; Lee et al., 2004); increases in serum creatinine observed with cimetidine; immune-mediated interstitial nephritis; both cholestatic and hepatocellular injury (Fisher and Le Couteur, 2001); CNS side effects including confusion, restlessness, somnolence, agitation, headaches, dizziness, and hallucinations and seizures with prolonged therapy (Cantu and Korek, 1991); and B12 deficiency with long-term H2RA use (Lam et al., 2013); however, many of these adverse effects are rapidly reversible or resolve after withdrawal of the drug (Fisher and Le Couteur, 2001). In our study, we found a lower PPI-related asthma risk if the patients used the H2RA first, before PPIs use.

Some past studies have found an association between GERD and asthma; however, no causal link between GERD and asthma was evident in our study. Conversely, we found that PPIs, rather than GERD, influence the subsequent occurrence of asthma. Our results showed that most PPIs were associated with asthma risk; the exception was rabeprazole. Our smaller patient sample size is a likely explanation, but further study could be considered to evaluate this difference.

Some limitations of this study should be considered. First, we could not establish a causal relationship between PPIs and asthma risk based on this retrospective cohort study. A prospective double blind study would be more convincing. Second, we found a higher incidence of asthma for the patients without peptic ulcer or exposure to asthma-related medications (NSAIDs and acetaminophen) in the PPI group than in the non-PPI group. A well-designed randomized control trial is necessary to confirm the higher asthma risk of PPIs, but PPIs are more popular regimens clinically due to their strong antacid effects and relatively low cost. Third, we noticed that rabeprazole did not increase the asthma risk, in contrast to the other PPIs (esomeprazole, lansoprazole, omeprazole, and pantoprazole). We are unable to explain this difference, but it might reflect the lower use of rabeprazole in our study population or, indeed, a pharmacological difference.

## Conclusions

Our data suggest that patients with long-time PPI use have a significantly higher risk of developing asthma when compared with the general population, regardless of gender and age. We recommended H2RA use prior to PPIs in patients who have no underlying peptic ulcers. The pathophysiological association between PPIs and asthma needs further investigation.

## Data Availability Statement

All datasets generated for this study are included in the article/supplementary material.

## Ethics Statement

The studies involving human participants were reviewed and approved by institutional review board of Chung Shan Medical University Hospital. Written informed consent for participation was not required for this study in accordance with the national legislation and the institutional requirements.

## Author Contributions

Conception and design: Y-TW and JW. Administrative support: JW. Data collection and organization: All authors. Data analysis and interpretation: All authors. Manuscript writing: All authors. Final approval of the manuscript: All authors.

## Funding

This work was supported by grants from Chung Shan Medical University Hospital research program, Taichung, Taiwan (CSH-2018-C-023).

## Conflict of Interest

The authors declare that the research was conducted in the absence of any commercial or financial relationships that could be construed as a potential conflict of interest.

## Abbreviations

PPIs, proton pump inhibitors; GERD, gastroesophageal reflux disease; H2RA, histamine 2 receptor antagonists; NSAIDs, nonsteroidal anti-inflammatory drugs.
